# Non-Invasive Respiratory Volume Monitoring to Detect Apnea in Post-Operative Patients: Case Series

**DOI:** 10.14740/jocmr1718w

**Published:** 2014-03-31

**Authors:** Christopher Voscopoulos, Diane Ladd, Lisa Campana, Edward George

**Affiliations:** a Department of Anesthesiology, Perioperative and Pain Medicine, Brigham and Women’s Hospital, Harvard Medical School, Boston, MA, USA; b School of Nursing, West Virginia University, Morgantown WV, USA; c Respiratory Motion, Inc., Waltham MA, USA; d Department on Anesthesia, Massachusetts General Hospital, Harvard Medical School, Boston, MA, USA

**Keywords:** Non-invasive, Respiratory, Volume, Monitor, Apnea, Ventilation, Post-operative, Quantitative

## Abstract

Obstructive sleep apnea (OSA) is a potential independent risk factor for postoperative complications, adverse surgical outcomes, and longer hospital stays. Obese patients with OSA have increased post-operative complications. An estimated 25-30% of pre-operative patients are at a high risk for OSA. A novel, non-invasive respiratory volume monitor (RVM) has been developed to provide a real time respiratory curve demonstrating lung volumes as well as a continuous, display of minute ventilation, tidal volume and respiratory rate. Clinical application of this device in the post-anesthesia care unit (PACU) can “unmask” post-operative apneic events resulting from partial or complete airway collapse due to the residual effects of narcotic administration and volatile and/or intravenous anesthetics. Clinical examples from two patients, one with known OSA and one without a previous diagnosis of OSA, monitored in the PACU with RVM are presented here. Post-operatively both patients had an increase in apneic episodes with significant decreases in their MV during apneic episodes after opioid administration as compared to pre-op baseline. In addition, oxygen saturation, for both patients, which is an essential component of current respiratory monitoring remained normal in the cases presented, despite the significant decreases in MV. Continuous RVM monitoring demonstrates both changes in respiratory patterns and overall adequacy of ventilation, and allows practitioners to quantify the increase in the number and duration apneic episodes as a response to narcotic administration. These case studies demonstrate that a non-invasive respiratory volume monitoring system can detect and quantify respiratory disturbances that currently go undetected.

## Introduction

Previous studies have established obstructive sleep apnea (OSA) as an independent risk factor for postoperative complications, adverse outcomes, and longer hospital stays [1-3]. Patients with OSA have an increase in post-operative complications, such as: oxygen desaturation, atelectasis, and increased pain [4]. OSA remains underdiagnosed with an estimated 25-30% of pre-operative patients at a high risk for OSA [5].The standard measure of OSA is the apnea-hypopnea index (AHI): the number of apneas and hypopneas per hour of sleep [6]. The sleep monitoring equipment required to calculate AHI is not generally available outside of the laboratory setting. Standard polysomnography (PSG) data acquired in the sleep laboratory does not assess the effects of opioids or supplemental oxygen, which can dramatically alter respiratory physiology and are commonly used in the post-operative setting. Therefore it is not readily translatable to the postoperative setting where oxygen therapy and the use of opioids for pain management are ubiquitous. Further, the AHI is mostly suitable for post-hoc analysis, rather than real-time, actionable, clinical, point-of-care decision-making. These deficiencies are the likely cause of the lack of correlation between pre-operative OSA severity and post-operative respiratory compromise that has been demonstrated in several studies [7-11]. OSA events may be more frequent or severe post-operatively than demonstrated in the sleep laboratory [2, 7-11]. These “unmasked” post-operative apneic events can result from partial airway collapse due to the residual effects of narcotic administration and volatile and/or intravenous anesthetics [12, 13], making adequate respiratory monitoring in the post-anesthesia care unit (PACU) even more essential.

In the PACU, conventional respiratory monitoring and subjective clinical assessment lack the ability to detect and quantify apneas in real-time, and as a result, patients may go untreated. Oxygen desaturation, which can result from prolonged or frequent apnea, may be masked by the use of supplemental oxygen; clinical observation data are intermittent and subjective, and capnography has challenges with implementation and consistency of use. Until recently, there has been no continuous, non-invasive, objective method for measuring disturbances in breathing patterns in non-intubated patients and quantify the adequacy of ventilation. This has complicated attempts to determine the full scope of post-operative airway obstruction. A non-invasive respiratory volume monitor (RVM) (ExSpiron, Respiratory Motion, Inc.) has been developed to provide a real time respiratory volume curves accompanied by a continuous, display of minute ventilation (MV), tidal volume (TV) and respiratory rate (RR). This FDA approved impedance based RVM system has been demonstrated to provide accurate measurements of MV, TV and RR in non-intubated patients [14, 15]. Previous work has shown that opioid use in patients with MV less than 80% predicted values could lead to marked decrease to levels below 40% predicted [16, 17]. The RVM curve can show respiratory patterns and real-time decreases in MV that can be used as a proxy for apneic breathing patterns.

Two cases are reported here, showing RVM use in the PACU. One patient had a pre-operative diagnosis of OSA and the other one did not. Permission was obtained from both patients for publication.

## Case Reports

In the following cases MV, TV and RR measurements were derived from continuous respiratory volume curves (30 second averages updating every 5 seconds). Apneic events were defined as episodes with no detected breaths lasting at least 10 seconds, and hypopneic events were defined as episodes lasting at least 10 seconds with a tidal volume reduction of at least 50% from baseline. MV, TV, and RR were calculated during apneic and non-apneic periods. A respiratory disturbance index (RDI) was also calculated and defined as the number of apneic and hypopneic episodes per hour.

### Case 1

Patient A: 79-year-old male, 100 kg, 168 cm (BMI = 35kg/m^2^) underwent knee replacement surgery under regional anesthesia. Past medical history was positive for OSA, asthma, and chronic obstructive pulmonary disease. Prior to the procedure, the patient received a single injection femoral nerve block and spinal anesthesia. Pre-operatively, 50 μg of fentanyl and 1 mg of midazolam were administered intravenously and the patient received an additional 15 μg fentanyl, 262 mg propofol, and 2 mg midazolam intra-operatively. He received no additional opioids during his PACU stay. RVM data were collected for a total of 292 minutes (22 minutes pre-operatively, 132 minutes during surgery, and 138 minutes during recovery in the PACU). Vital sign measurements and pulse oximetry data were also obtained. Supplemental oxygen was supplied (2 L/min via nasal cannula) during the PACU stay.

**Figure 1:**
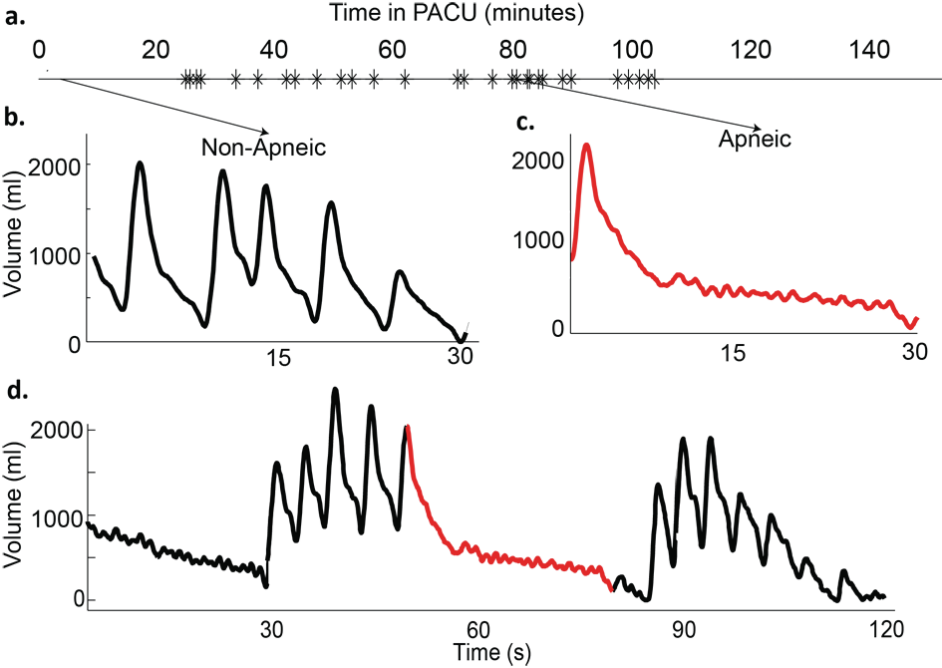
Example traces from patient A. a)Timeline of patient’s stay in the post-anesthesia care unit (PACU), with each apnea/hypopnea plotted; b) 30 Second trace of normal breathing pattern (minute ventilation (MV] = 14.2 L/min, RR = 11, tidal volume (TV) = 1.3 L); c) 30 second trace from an apneic time period (MV = 2.4 L/min, respiratory rate (RR) = 2, TV = 1.2 L); d) 120 second trace from a period with repetitive apneas. (MV = 5.9 L/min, RR = 6, TV = 0.99 L).

Using a standard formula based on body surface area (BSA) for males (BSA × 4), patient A’s predicted MV (sufficient to maintain blood oxygen and carbon dioxide levels under baseline conditions) was calculated to be 8.3 L/min. His measured MV, recorded by the RVM prior to surgery during quiet breathing, was 15.1 ± 0.6 L/min (mean ± SEM), likely due to anxiety during the pre-operative period. He had 3 episodes of hypopnea noted in the 22 minutes of pre-operative RVM recordings after the administration of 1 mg of midazolam prior to his femoral block. Post-operatively, he had 29 apneic and hypopneic events, averaging 17 ± 4.9 sec in length during his PACU stay (Fig. 1a). During one episode of depressed breathing, his MV decreased to 2.4 L/min, equivalent to 29% of his predicted MV and 16% of his pre-operative MV (Fig. 1c). Although recovery breaths following apneic episodes were on the order of 1L (Fig. 1d), they did not sufficiently restore MV to baseline levels (MV = 5.9 L/min, 40% of pre-op baseline). The apneic events were concentrated over a period of 78 minutes (RDI = 22 events/hour) during the time that the patient was observed to be dozing on and off.

**Figure 2:**
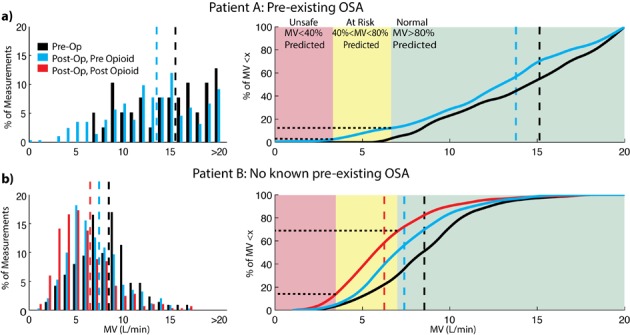
(a, b) Distribution of minute ventilation (MV) in both patients. Histograms (left panels) show the fraction of MV measurements at a particular MV level prior to surgery (black) and after surgery (blue - before opioid and red - after opioid). The cumulative distributions (right panels) show the fraction of measurements below a given MV level during a given period of time (black - pre-op, blue - post-op pre-opioid, and red - post-op post-opioid). In patient A (top panels), average MV drops from 15.1 to 13.8 L/min and 10% of the time post-operatively the patient’s MV is below 80% predicted, in the “at risk” zone (yellow). In patient B (bottom panels), the average MV drops from 8.4 L/min pre-operatively to 7.4 L/minpost-operatively (blue) and further drops to 6.3 L/min after the administration of the opioid (red). Post-opioid, patient B spendsnearly 13% of the time with MV below 40% of predicted in the “unsafe” zone (red).

Pre-operatively and post-operatively, the RVM measured a natural variation in his MV. A systematic post-hoc analysis found that pre-operatively MV ranged from 7.2 to 23.8 L/min with an average of 15.1 L/min and 100% of all readings were above 80% of this patient’s predicted MV (6.6 L/min) (Fig. 2a). Post-operatively, the distribution of MV measurements was drastically shifted. While the average MV (13.8 L/min) remained above predicted (8.3 L/min), the range of recorded MV was from a dangerously low level of 0.4 L/min during periods of apneic episodes to 28.0 L/min. A histogram, showing the relative distribution of measured MV both pre-operatively (black) and post-operatively (blue), is shown in Figure 2a, left. The systematic change in MV is better visualized as a shift in the cumulative distribution function (CDF) of MV values from the pre-operative (Pre-op) to post-operative (Post-op) periods (Fig. 2a) (right). These CDFs plot the fraction of time when his MV was less than or equal to a particular MV measurement. Pre-operatively, 100% of MV measurements were greater than 80% predicted. Post-operatively, 10% of the patient’s MV was below 80% predicted (“at risk” zone) and 1% of the time it was below 40% predicted (“unsafe” zone). Pulse oximetry data were recorded during the PACU stay and the oxygen saturation never fell below 95%, he received oxygen (2 L via nasal cannula); there was no indication of apneic breathing.

### Case 2

Patient B: 73-year-old male, 94 kg, 183 cm (BMI = 28 kg/m^2^) underwent knee replacement surgery under regional anesthesia. Past medical history was negative for OSA, or any other chronic respiratory conditions. Before the procedure, the patient received a single injection femoral nerve block and spinal anesthesia. Pre-operatively, 50 μg of fentanyl and 1 mg midazolam were administered intravenously. Intra-operatively, the patient received intravenously an additional 125 μg fentanyl, 135 mg propofol, and 1.5 mg midazolam. RVM data were collected for 558 minutes (111 minutes pre-op, 115 minutes during surgery, and 332 minutes in the PACU). Vital sign and oxygen saturation measurements were also obtained. The patient entered the PACU on 6 L/min of O_2_ via face mask but was switched to room air after 15 minutes. One hundred and seventy five minutes after arrival in the PACU, the patient received 2 mg of morphine intravenously.

**Figure 3:**
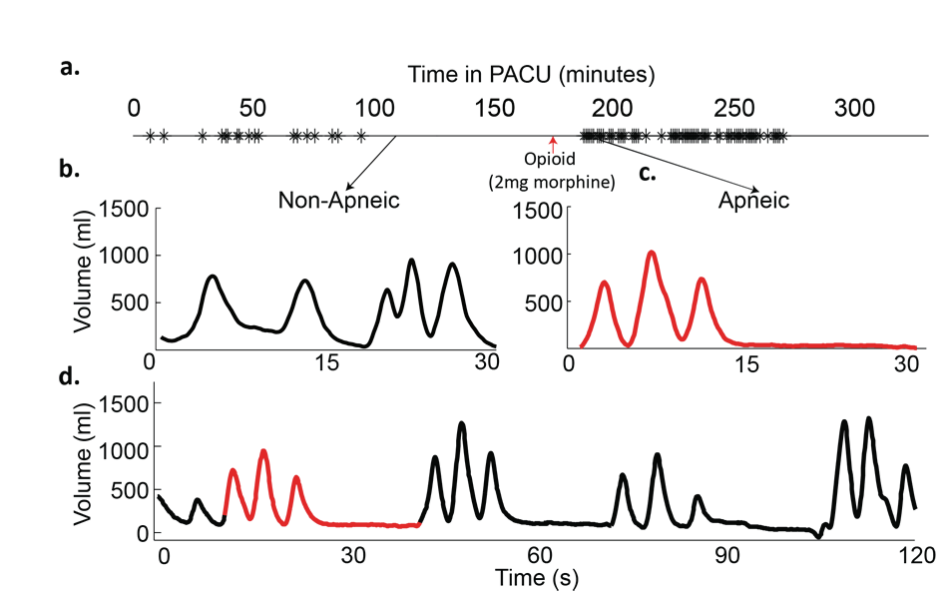
Example traces from patient B. a) Timeline of patient’s stay in the post-anesthesia care unit (PACU), with each apnea/hypopnea plotted; b) 30 Second trace of normal breathing pattern before opioid administration (minute ventilation (MV) = 8.2 L/min, RR = 11, TV = 730 m); c) 30 second trace from an apneic time period after opioid administration (MV = 4.5 L/min, RR = 6, TV = 750 mL); d) 120 second trace from a period with repetitive apneas. (MV = 4.7 L/min, RR = 6.5, TV = 730 mL).

Based on the BSA formula, his predicted MV was 8.7 L/min, consistent with his MV measured prior to surgery (8.4 ± 0.2 L/min, mean ± SEM). He had no episodes of apnea noted in the 111 minutes of RVM recording before surgery. Post-operatively, he had 88 apneic events occurring over 5.5 hours in the PACU (RDI = 16 events/hour). He had two distinct time periods where repetitive apneas occurred: one prior to the administration of 2 mg morphine and one after the morphine was administered (Fig. 3a). During apneic breathing episodes his MV was reduced to 4.5 L/min, 54% of his pre-operative MV (Fig. 3c). Similar to patient A, recovery breaths were not large enough to compensate for the apnea and return MV to baseline levels (Fig. 3d) (MV = 4.7 L/min, 56% of pre-op). Prior to opioid dosing, 18 apneic events occur over 1.5 hours (RDI = 12 events/hour) with an average length of 14 ± 4 sec. After administration of a single small dose of opioid, the apneic events became more frequent and longer in duration and snoring was observed. Seventy events occurred in the 1.4 hours post-opioid (RDI = 50 events/hour) with an average apnea length of 18 ± 5 seconds.

Similar to patient A, the pre-operative RVM measured MV ranged from 2.6 to 17.7 L/min with an average of 8.4 L/min (Fig. 2b) (left). Over 70% of pre-operative MV measurements were above 80% and 98% were above 40% of this patient’s predicted MV (Fig. 2b) (right). Post-operatively, the average MV was decreased from preoperative levels to7.4 L/min before the opioid was given and dropped further to an average of 6.3 L/min after the opioid was given, where MV fell to as low as 1.0 L/min during periods containing apneic episodes. Postoperatively in the 175 minutes before the opioid was given in the PACU, 51% of the time patient B’s MV was below 80% predicted and 4% of the time below 40% predicted MV. In the 157 minutes after opioid administration prior to leaving the PACU, 63% of the time his MV was below 80% predicted and 13% of the time was below 40% predicted. Thirteen percent of the time his MV was below 40% of predicted, post-opioid, compared to 4% of the time pre-opioid and less than 2% of the time pre-operatively. The cumulative distribution of MV values for patient B during pre-op, post-op pre-opioid and post-op post-opioid periods are shown in Figure 2b, right. Pulse oximetry data were recorded during the PACU stay, but the oxygen saturation never fell below 95% and there were no indications that apneic breathing had occurred.

## Discussion

Improvements in respiratory monitoring in the PACU could enable clinical staff to accurately assess respiratory status, promoting timely interventions to address potential respiratory compromise. These case studies demonstrate how a non-invasive respiratory volume monitoring system can detect and quantify respiratory disturbances that currently go undetected. RVM provides both a real-time respiratory volume trace that displays both changes in respiratory pattern and episodes of true apnea, as well as a quantitative display of minute ventilation, an important metric of respiratory competence.

Oxygen saturation, which is an essential component of current respiratory monitoring, remained normal in the cases presented, despite noted decreases of MV down to less than 5% predicted (0.4 L/min) during apneic periods. This finding reinforces the fact that a decrease in measured oxygen saturation, especially in the presence of supplemental oxygen, is an insensitive indicator of respiratory status. Reduction in oxygen saturation occurs cyclically with apneic breathing and can easily be missed by current monitoring systems or be masked by oxygen administration. The RVM was not only able to measure the length and pattern of apneic episodes, but also quantify the increase in the number and duration of such episodes as a response to narcotic administration. This highlights the importance of respiratory monitoring in the post-operative environment where narcotics are frequently used to manage pain. RVM was able to quantify the adequacy of rescue breaths to maintain respiratory homeostasis. The post-hoc analysis demonstrates the utility of the RVM in comprehensive respiratory analysis, which may augment or supplant polysomnography. Patient B, non-obese and with no previous diagnosis of OSA, had substantively more respiratory depression postoperatively than patient A, with known OSA.

The 2011 American Society of Anesthesiologists’ guidelines require monitoring of not just oxygenation, but also ventilation in the PACU. The quantitative measurements of MV and TV from the RVM can assist in decision making improve patient safety and optimize pain management in patients with or at risk for OSA. The RVM can be used with other measures for the assessment of ventilation in non-intubated patients. Further studies to formalize the use of RVM for the detection and quantitation of apneas are on-going.
